# Design and Performance of Two‐Sided Self‐Protecting Perovskite Solar Cells for Indoor Vertical Applications

**DOI:** 10.1002/smsc.202500603

**Published:** 2026-03-19

**Authors:** Salvatore Valastro, Sebastian Ferranti, Rosa Previti, Michele Dellutri, Simone Galliano, Alessandra Alberti

**Affiliations:** ^1^ CNR‐IMM Catania Italy; ^2^ STMicroelectronics Srl Catania Italy; ^3^ Department of Chemistry NIS Interdepartmental Centre and INSTM Reference Centre University of Turin Turin Italy

**Keywords:** BAPV, bifacial, durability, low cost, power generator

## Abstract

We present a novel photovoltaic architecture that integrates two perovskite solar cells (PSCs), each fabricated on a separate substrate and bonded back‐to‐back, forming a self‐protective and bifacial device. This configuration, named two‐sided self‐protecting perovskite solar cell (2S‐SP PSC), replaces the traditional passive glass encapsulation with a second active PSC. In a 2S‐SP PSC, the active area for light harvesting doubles within a single‐device footprint without increasing the overall device thickness. The two PSCs are electrically connected in series or parallel configuration and are self‐encapsulated back‐to‐back using conventional materials that ensure mechanical integrity and further moisture protection. We realized demonstrators of the 2S‐SP PSC using fully printable, HTL‐free, mesoporous carbon‐based AVA‐MAPbI_3_ PSCs, an architecture well suited for the industrial production of photovoltaic panels. Compared to single monofacial counterparts, the 2S‐SP PSCs deliver nearly double the output in vertical configurations (24.6 μW vs 51.3 μW) under indoor artificial lighting (920 Lx, 6500 K). In addition, they exhibit long‐term stability due to the mutual encapsulation of the two cells. An engineered 2S‐SP mini‐module powering a humidity/temperature sensor under ambient indoor light further highlights the potential application in self‐powered IoT devices and building‐applied photovoltaics.

## Graphical abstract

Two‐sided self‐protecting perovskite solar cell scheme in a working ambient, doubling performances and extending stability over time with respect to the reference perovskite solar cell.

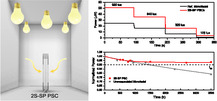

## 1 Introduction

Perovskite solar cells (PSCs) have recently surpassed the power conversion efficiency (PCE) record of traditional silicon solar cells, achieving an impressive 27.3% (NREL Efficiency Chart [1]). Beyond their conventional role as single‐junction solar cells, perovskites are increasingly integrated with silicon in tandem architectures [2]. PSCs are well‐suited for various applications, such as integration into buildings (building integrated photovoltaics – BIPV and building‐applied photovoltaics – BAPV) or use in the agrivoltaics sector [3], thanks to their semi‐transparent properties [4]. Another emerging application of PSCs is their use in powering Internet of Things (IoT) devices under indoor environments [5, 6, 7]. Compared to silicon solar cells, perovskites exhibit superior performance under indoor low‐light conditions, making them well‐suited for energy harvesting in ambient lighting [8]. They can efficiently power sensors – such as humidity and temperature sensors – as well as various smart home automation devices.

Despite these advancements, one of the primary challenges facing PSCs remains their stability. Perovskite materials are highly susceptible to environmental factors such as moisture, heat, and ultraviolet (UV) exposure [9], which can degrade their performance over time [10]. To mitigate this issue, effective encapsulation is crucial. Among various encapsulation techniques, cover‐glass encapsulation has been identified as one of the most effective strategies [11]. This method involves placing a protective glass layer over the PSC and sealing the device's surface with an encapsulant or adhesive, creating a barrier that prevents moisture ingress [12].The dual moisture‐impermeable surfaces formed by the cover‐glass significantly enhance the long‐term durability of PSCs compared to other encapsulation methods [13]. Additional edge sealing through specific materials further minimizes the moisture access into the device [14].

In addition to improving stability, enhancing the power output of PSCs remains a critical challenge. A promising and cost‐effective strategy for increasing power generation is the use of bifacial PSCs [15, 16]. Unlike monofacial PSCs, which capture light from only one side, conventional bifacial PSCs utilize transparent top and bottom electrodes, rather than opaque metal or carbon layers, allowing them to harvest both direct sunlight and reflected or diffuse light from surrounding surfaces [17].

For indoor photovoltaic (PV) applications, optimized bifacial single PSC designs have shown potential for significantly improving energy conversion in low‐light environments [18], particularly when deployed in vertical [19] or inclined configurations. This advantage makes them suitable for applications where space is limited, such as integrated PV in smart buildings and self‐powered electronic systems.

Although conventional semitransparent bifacial PSCs can collect light from both sides, their performance is constrained by the use of transparent electrodes, specifically the rear TCO, which cannot be annealed without damaging the perovskite stack, results in higher sheet resistance, stronger parasitic absorption, and poorer optical quality than in opaque‐ electrode monofacial PSCs. Consequently, these lead to electrical power losses, especially under high photocurrent conditions [17].

These optical and electrical losses also severely limit the bifaciality factor, which typically falls in the 0.6–0.8 range, meaning the rear side provides only 60–80% of the front‐side power output. Even when employing advanced transparent electrodes, such as single‐walled carbon nanotubes [20], ultrathin metal stacks (Au, Ag), or metal–oxide composite electrodes (TeO_2_/Ag/WO_*x*_, MoO_*x*_/Ag), the rear‐side performance remains lower [21] compared to the front side. Overall, these intrinsic optical and electrical asymmetries prevent a conventional semitransparent bifacial PSC from operating as the functional equivalent of two identical fully optimized opaque monofacial PSCs connected in parallel.

As with monofacial PSCs, cover‐glass encapsulation, along with appropriate sealants [22], is essential to maintain the long‐term stability and efficiency of bifacial devices. In addition, bifacial architectures demand a mandatory fully transparent encapsulant, whose non‐ideal optical properties or gradual degradation over time can give rise to additional optical losses on the rear side [12]. Addressing these stability, optical, and conductivity challenges is crucial for further advancing conventional bifacial perovskite solar cells as a viable high‐performance photovoltaic technology.

In this study, we introduce a novel bifacial PSC architecture, named two‐sided self‐protecting perovskite solar cell (2S‐SP PSC). This configuration overcomes key limitations of conventional bifacial PSCs by employing a mechanically stacked tandem of two PSCs that offers a versatile, effective solution to space use and stability. 2S‐SP PSC mainstays have been demonstrated for opaque PSCs and indoor applications; however, their implementation could also be broadened to include building‐integrated photovoltaics (BIPV), building‐applied photovoltaics (BAPV), and outdoor installations, such as inclined configurations designed to capture albedo light or vertical configurations suited for uniformly low‐light environments like urban canyons.

## 2 Results and Discussion

### 2.1 Two‐Sided Self‐Encapsulated Perovskite Solar Cells Architecture Concept

The proposed architecture implies two PSCs fabricated each on a substrate (in principle, glass or flexible foils) that are bonded at the side of the top electrode through an encapsulant sealer. They are thus inherently and mutually self‐protected against humidity or other external agents. In 2S‐SP PSCs, the innovation consists of replacing the passive cover glass of a traditionally encapsulated PSC architecture with a second active PSC. The two PSCs are electrically connected in a two‐terminal (2T) configuration, with the cells in series or parallel to increase the voltage or the current output, respectively. We note that a four‐terminal (4T) configuration is also feasible.

In a 2S‐SP PSC, the active area for light harvesting doubles within a single‐device footprint without increasing the overall device thickness. The two device components are also long lasting due to the mutual protection and a common encapsulant. Another key advantage is that any PSC layout is compatible with this 2S‐SP PSC scheme, and a semitransparent device is not strictly required.

**FIGURE 1:**
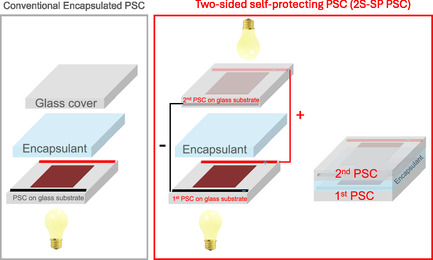
Scheme of a conventional encapsulated perovskite solar cell vs a two‐sided self‐protecting configuration.

In Figure 1, a case study of 2S‐SP PSC configuration is shown with two opaque PSCs connected in parallel: both sides of the solar cell capture light and convert it into electricity, leading to a two‐fold increase in the generated power, due to the additive contribution of the two independent devices. Simultaneously, the external glass substrates of each device provide mutual protection from environmental humidity [23]. An encapsulant interlayer is also used in between the two devices to provide further protection.

The synergy between bifacial design and self‐encapsulation boosts performance and device durability. The proposed 2S‐SP PSC configuration applies, in principle, to all perovskite solar cell architectures (including n‐i‐p, p‐i‐n [24, 25], mesoporous‐based, single crystal‐based, and HTL‐free structures), to any perovskite composition deposited with any kind of preparation procedure (including solution processing [26] and HV‐PVD [27], LV‐PSE [28]) and to any HTL/ETL materials. The two PSCs in the proposed 2S‐SP PSC configuration can be fabricated 1) identically, meaning the same architecture and the same perovskite composition, 2) similarly, using the same device architecture but different perovskite compositions, and 3) similarly, using the same perovskite composition but different architecture, depending on the specific application. In a 2T‐configuration, where the cells are in parallel or series, it is crucial that the working voltage, typically selected near the maximum power point (MPP), is matched between the two PSCs to avoid electrical and power losses. For indoor applications with only artificial lighting, this condition is easily achieved due to the uniform light distribution, which allows similar light conditions when two identical PSCs are used. In contrast, for BIPV or BAPV, or outdoor application in inclined configuration to absorb the albedo light, the two PSCs often experience differing light intensities, leading to variations in open‐circuit voltage (V_OC_), fill factor (FF), and short‐circuit current density (J_SC_). These discrepancies result in distinct MPP voltages (V_MPP_), potentially requiring different operating biases for each solar cell. Notably, the V_MPP_ of a PSC exposed to direct sunlight is typically higher than that of a PSC illuminated by indoor or diffuse light. This mismatch can lead to performance losses, as the cell under lower illumination may behave more like a load than a generator within the circuit (Figure S1). This occurs when the fixed operating voltage exceeds the V_OC_ of the less‐illuminated PSC, inducing a reverse current that opposes the photogenerated current. The extent of this mismatch is influenced by defect‐assisted recombination within the devices [29]. In such scenarios, the series resistance (e.g., of the top electrode) and the cell geometry (e.g., active area) become critical design parameters and can be optimized to align V_MPP_ values under both 1 sun and indoor lighting. Alternatively, voltage matching and spectral optimization can be achieved by employing different perovskite compositions for each PSC, using a wider bandgap formulation for indoor conditions and a narrower bandgap for outdoor exposure [7]. As a further solution, 4T‐configuration could be applied in order to allow the two cells to operate at their own MPP independently.

The encapsulant material can be integrated either as a laminated foil or as a liquid polymer [22] applied through drop‐casting, spin‐coating, or blade coating, provided the deposition method is compatible with the perovskite and other cell materials regarding temperature, pressure, solvent compatibility, and related parameters. Conventional encapsulation materials [30]. commonly utilized in perovskite solar cells, such as ethylene‐vinyl acetate, polyurethane, and polyolefin, are suitable options, and additional interlayers, such as Kapton tape, may also be introduced between the encapsulant and the two PSCs. Edge sealing of the device with materials like polyisobutylene (PIB) [31] is also feasible in this architecture.

Potential indoor applications include vertically installing the 2S‐SP PSC on horizontal surfaces such as desks and shelves, as well as on vertical elements like window frames (BIPV), doors with glass panels, and interior walls (BAPV). For the latter, a small spacing from the mounting surface is required to ensure that both faces of the device can receive direct, diffuse, and reflected illumination. To evaluate the practical benefit of the vertical bifacial configuration under realistic indoor conditions, we performed detailed lighting simulations using DIALux evo. In a typical office room, when the 2S‐SP device is placed vertically in a room corner at 240 cm height, at a distance of 30 cm and angled 45° with respect to the surrounding walls, the two faces receive 364 Lx (front, directly illuminated) and 264 Lx (rear, reflected and diffused from adjacent walls), resulting in a total of 628 Lx (Figure S2). A vertical monofacial device in the same position would collect only the 364 Lx incident on the front side, and a horizontal monofacial device at the same location would receive 274 Lx. Thus, the vertical bifacial configuration enhances usable illuminance by 72.5% compared to a vertical monofacial device and 129% compared to a horizontal monofacial device.

We further simulated integration on a white office desk (90% reflectance. 75 cm from the floor). Depending on the module position and orientation, the total vertical illuminance on the two faces of the 2S‐SP PSC increases by 13.7–59.2% relative to the horizontal monofacial case (Figure S3‐S4). This improvement arises from diffuse and reflected illumination of the walls, an effect particularly pronounced in room regions not directly under the LED light source, where indirect light dominates.

These simulations indicate that, in many realistic indoor applications, such as on desks and corners, the bifacial architecture in vertical configuration enables higher light harvesting per footprint by capturing illumination components that opaque monofacial modules cannot absorb.

For these applications, a cost‐effective and easily scalable architectural design is highly desirable.

### 2.2 Two‐Sided Self‐Encapsulated HTL‐Free C‐Based Perovskite Solar Cells Architecture

To prove the feasibility and advantages of the proposed architecture, we fabricated a two‐sided self‐protecting PSC by using two identical HTL‐free mesoporous carbon‐based perovskite solar cells (mC‐PSCs) with integrated AVA‐MAPbI_3_ [32]. The two mC‐PSCs are bonded using a foil composed of a blend of polyolefin and paraffin wax, which is vacuum laminated at 70°C, with a layer of Kapton tape used as an interlayer, which was proven to improve the stability over the time for this kind of devices [22, 33]. No edge sealing is employed. The impact of the encapsulation process on the J–V curves is shown in Figure S5 and is consistent with the behaviour typically observed during conventional cover‐glass encapsulation [22, 34]. The contacts are externally extended to enable electrical measurements from the device side to the glass side using silver paste tracks. The two mC‐PSCs are electrically connected in parallel for indoor application [35] in vertical configuration. The device active area is 1.5 cm^2^. The use of mC‐PSC components is justified by three main advantages: (1) they are fully printable, which enhances its industrial appeal through scalable and cost‐efficient manufacturing[36]; (2) they are cost‐effective without expensive HTLs such as spiro‐MeOTAD and metallic top electrodes like gold and silver [37]; and (3) they offer high operational stability [38, 39]. Importantly, the low cost is particularly relevant for indoor use, where economic competitiveness is a key determinant for commercial adoption. As for the use of AVA‐MAPbI_3_, it is stable under stress tests such as damp heat test (85°C/85%RH, 1,100 h), thermal cycling test (‐40° ∼ 85°C, 200 cycles), and MPPT light soaking test, with AVA that prevents MAI loss, suppresses crystal reconstruction, and inhibits irreversible ionic migration^33^. Moreover, a recent study [40] demonstrated impressive stability of MAPbI_3_ achieved through rapid crystallization, reestablishing it as a promising candidate among photovoltaic technologies. Although the bandgap of AVA‐MAPbI_3_ (1.61 eV)^33^ is lower than the optimal value of 1.9–2.0 eV for indoor applications [41], recent studies have demonstrated that materials with lower bandgaps can still achieve good performance under indoor lighting conditions [42].

**FIGURE 2:**
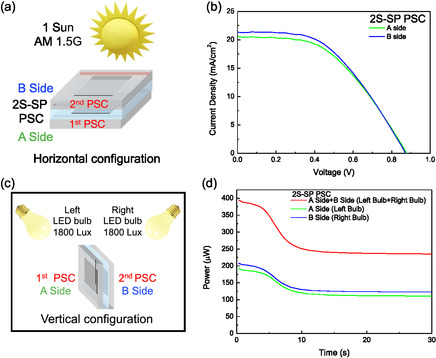
(a) Scheme of 2S‐SP PSC under 1 Sun (AM 1.5G) illumination. (b) J–V curves of side A and side B of 2S‐SP PSC under 1 Sun AM1.5G used as reference. (c) Scheme of 2S‐SP PSC under indoor illumination (two LED bulbs 6500 K) at 1800 Lx each, illuminating the two‐sides. (d) Generated power at the maximum power point of the 2S‐SP PSC in vertical configuration under indoor illumination (two LED bulbs 6500 K) at 1800 Lx each, illuminating the two‐sides.

The bifacial capability of the 2S‐SP PSCs is initially demonstrated by illuminating each side of the device separately, while keeping the opposite side shaded. The test were done with: 1) the 2S‐SP PSC placed in horizontal configuration under a Solar simulator (1 Sun, AM 1.5G) (Figure 2a), used as a reference and 2) the 2S‐SP PSC placed in vertical configuration illuminated by two White LED bulbs (1800 Lx each‐6500 K) inside a black‐painted box to avoid diffused light and to simulate uniform indoor conditions (Figure 2b). Each LED bulb is directed at a 45° angle toward only one face, allowing separate illumination of each side of the 2S‐SP PSC. The light spectrum of LED bulb is shown in Figure S6. Under 1 Sun illumination, the J–V curves acquired from Side A and Side B of the 2S‐SP‐PSC are similar, with a PCE of 8.61% for side A and 8.93% for side B (Figure 2c), demonstrating an almost perfect bifaciality of the 2S‐SP PSCs. This efficiency value well aligns with those reported in the literature for the same kind of devices [43]. Because the carbon electrode is fully opaque, each PSC is illuminated exclusively through its own glass side, with no optical cross‐talk between the two devices, as confirmed experimentally (see Figure S7 in the supporting information). No light is transmitted through the carbon electrode of the first PSC to reach the second PSC and thus, from an optical perspective, each device operates as an independent single‐junction PSC. In vertical configuration, the left bulb illuminates only the first PSC (A Side), while the right bulb illuminates only the second PSC (B side) (Figure 2b). As shown in Figure 2d, when a single bulb is on, only one side of the 2S‐SP PSCs is active, producing ~125 μW of stabilized power (at fixed voltage corresponding to the MPP of 0.48 V). However, when both bulbs are on, both sides are illuminated, resulting in a total stabilized power output of ~250 μW, equivalent to the combined output of the two individual PSCs.

### 2.3 Two‐Sided Self‐Encapsulated HTL‐Free C‐Based Perovskite Solar Cells for Indoor Applications

**FIGURE 3:**
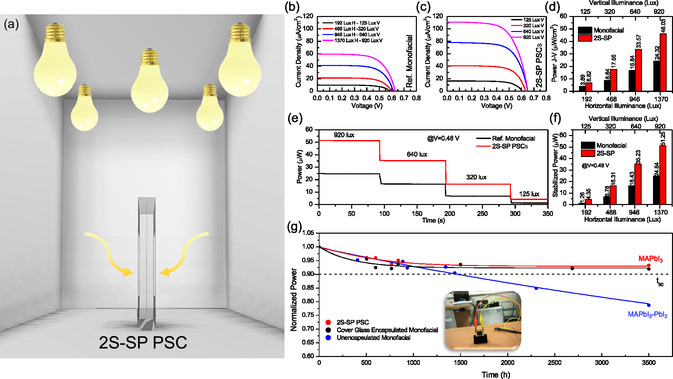
Scheme of the indoor measurements inside the white‐painted walls box with 5 LED bulbs with the 2S‐SP PSC in vertical configuration. (b) J–V curves at different illuminance conditions for the reference monofacial and (c) for the 2S‐SP PSC in vertical configuration. (d) Maximum power extracted from J–V curves at different illuminance conditions for monofacial and 2S‐SP PSC in vertical configuration. (e) Power at constant voltage (0.48 V) over the time at different illuminance conditions for monofacial and 2S‐SP PSC in vertical configuration. (f) Stabilized power at 0.48 V (V_MPP_) at different illuminance conditions for monofacial and 2S‐SP PSC in vertical configuration. (g) Normalized power over the time of the reference unencapsulated monofacial mC‐PSC, passive cover‐glass encapsulated PSC and 2S‐SP PSC, all stored in indoor conditions (30‐65% RH and 25 °C). The lines are guides for the eyes.

The device was thus tested in horizontal and vertical configuration in a box of 5 LED lamps of 6500 K with white‐painted walls to simulate the indoor illumination conditions at different illuminance conditions (Figure 3a). In particular, it was tested using a horizontal illuminance of 1368 Lx, 468, lux, 946 Lx, and 192 Lx, which in our experimental configuration corresponds to a vertical illuminance of 920, 640, 320, and 125 Lx. The last values are the effective values of illuminance that hit the sample in vertical configuration from both sides at the floor level, measured by positioning the luxmeter vertically towards both sides of the 2S‐SP PSC and summing the readings. These values rise when the device is moved closer to the LED lamps, reflecting the higher illuminance at shorter distances, with a logarithmic dependence, as shown in Figure S8. A conventional monofacial HTL‐free mesoporous carbon perovskite solar cell encapsulated with a cover glass is used as a reference. In vertical configuration, the analysis of J–V curves under varying illuminance values in the range 920–192 Lx (Figure 3 b–c), along with the extracted electrical power values (Figure 3d) and stabilized power output at a fixed voltage of 0.48 V, which is the V_MPP_ extracted from J–V curve) (Figure 3e‐f), demonstrates that the 2S‐SP PSC produces approximately twice the power output of a monofacial PSC. This increase is due to a twofold enhancement in photogenerated current ascribable to the bifacial property of the proposed architecture. In the case of the monofacial PSC, light harvesting from the LED source is possible exclusively through the glass side, as the carbon side is opaque and thus unable to contribute to light absorption. For instance, at a vertical illuminance value of 920 Lx, the monofacial PSC generates 24.64 µW, while the 2S‐SP PSC yields 51.3 µW (Statistics on 4 devices is shown as boxplots in Figure S9). The 2S‐SP PSC generated power is stable over a period of 16 h (Figure S10). The perfect bifaciality was further confirmed inside the 5 LED lamps box since the measurements performed on side A (while obscuring side B) and on side B (while obscuring side A) in horizontal configuration shows identical power output (Figure S11). The photovoltaic parameters values are shown in table S1, showing an efficiency extracted from J–V curves of around 18% for both sides of the 2S‐SP PSC at different illuminance conditions, with a stabilized efficiency of around 12%. The mismatch between the efficiency value calculated from J–V curves and the stabilized efficiency at constant voltage is due to ionic migration, in particular, to the cation displacement (depletion) occurring at the c‐TiO_2_ interface which strongly affects the electrons and holes distributions over time, as previously shown^33^. Efficiency values well align with those previously reported in the literature for the same AVA‐MAPbI_3_ mC‐PSC^36^. Comparable results are achieved using 3000 K lamps (spectrum shown in Figure S12 and performances in Figure S13), which are better suited for domestic applications. To demonstrate the effectiveness of the self‐protection of the 2S‐SP PSCs, we monitored the power output of the device over time compared to the unencapsulated and cover‐glass encapsulated monofacial device stored in indoor conditions (30–65% RH and 25°C). As illustrated in Figure 3g, the power of the unencapsulated PSC monotonically decreases reaching 78.7% of its initial value after 3500 h under ambient conditions due to moisture‐induced degradation of the perovskite layer (partial formation of PbI_2_) [22]. In contrast, the efficiency of 2S‐SP PSC stabilizes at 93.2% of the initial power output from 900 h up to at least 3500 h, similar to the passive cover‐glass encapsulated monofacial PSC (92.2%), highlighting the effectiveness of this configuration in preserving the perovskite photoactive phase by likewise doubling the generated power. To widely cover nature and fabrication method for the encapsulant, we alternatively used polyurethane resin (PU), applied in liquid form by drop‐casting, as reported in ref. [22] (see Experimental Section for details). In this configuration, Kapton tape was not used. The J–V characteristics of the 2S‐SP PSC and the reference monofacial device, shown in Figure S14, are consistent with the results obtained using laminated foil, confirming a twofold increase of the generated power together with an enhanced stability.

### 2.4 Two‐Sided Self‐Encapsulated HTL‐Free C‐Based Perovskite Solar Mini‐Module

**FIGURE 4:**
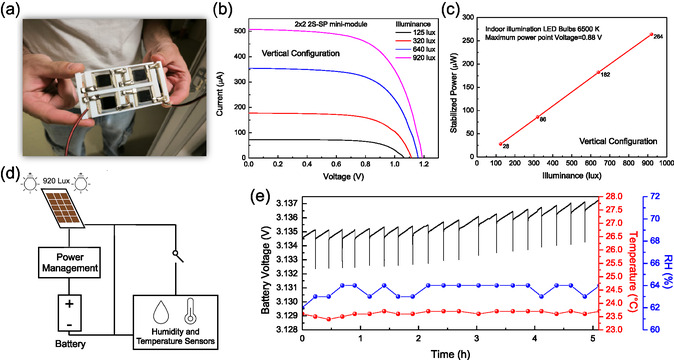
(a) Photo of the two‐sided self‐encapsulated HTL‐free C‐based perovskite solar mini‐module. (b) J–V curves at different illuminance conditions for 2S‐SP mini‐module in vertical configuration. (c)Stabilized power at 0.88V (V_MPP_) at different illuminance conditions for 2S‐SP mini‐module in vertical configuration. (d) Scheme of the simplified proof‐of‐concept circuit designed to power a board equipped with a humidity and temperature sensor. (e) Battery voltage measured temperature and humidity over time.

A 2x2 mini‐module (total active area = 6 cm^2^) was fabricated by integrating multiple bifacial double PSCs, as illustrated in Figure 4a. This module consists of a support frame with multiple through‐housing seats, each accommodating a 2S‐SP PSC. Each solar cell is positioned within its designated housing seat and electrically connected in a combination of two in series and two in parallel. As expected, the J–V curves measured under varying indoor illuminance demonstrate that J_SC_ and V_OC_ are approximately doubled compared to a single 2S‐SP PSC, as shown in the J–V Curves in Figure 4b. Consequently, the stabilized power output is nearly four times higher than that of a single 2S‐SP PSC (Figure 4c). This minimodule was ultimately integrated into a simplified proof‐of‐concept circuit designed to power a board equipped with a humidity and temperature sensor (Figure 4d). The circuit incorporates a power management system that regulates voltage to maximize energy harvested from the 2S‐SP minimodule, illuminated by a continuous light of 6500 K LED bulbs at 920 Lx (Figure S15‐S16), which subsequently charges a Li‐Po battery. A switch allows for periodic recording of humidity and temperature data at set intervals: when open, the 2S‐SP minimodule charges the battery; when closed, the battery powers the board for approximately 5 s to acquire temperature and humidity readings. We selected a reasonable interval of 15 min. Battery voltage measurements taken over 5 h show that at this interval, the battery can maintain its charge and even recharge, as indicated by the upward trend in voltage over time (Figure 4e). To further validate the system under realistic operating conditions, we performed an additional experiment alternating 1 hr light / 1 hr dark cycles, which revealed a clear stepwise charging behaviour of the battery with minimal voltage decrease in the dark (Figure S17). In addition, these results suggest an even more favourable trade‐off between overall charge and discharge under a more practical 16 h light / 8 h dark indoor daily cycle, as the light‐to‐dark time ratio is doubled compared with the conditions of the present experiment. These results suggest that the system can collect temperature and humidity data at shorter intervals, while the output power can be enhanced by increasing the number of solar cells in the minimodule; furthermore, optimization for IoT applications could be achieved by replacing the battery with supercapacitors and integrating communication technologies such as LoRa or bluetooth low energy (BLE).

## 3 Conclusions

This work introduces and experimentally validates the concept of 2S‐SP PSC, a design that combines bifacial light harvesting and encapsulation for performance durability into a single technological solution. By replacing the passive encapsulation layer with a second active PSC, the proposed architecture doubles the effective power output per area, particularly in inclined or vertical configurations, relevant for indoor, outdoor, and building‐applied photovoltaics. Comparative measurements with conventional monofacial PSCs encapsulated with cover‐glass demonstrate that the enhanced power generation arises from the simultaneous harvesting of direct, diffuse, and reflected light by two photovoltaic sub‐cells operating as optically independent energy generators, with no optical cross‐talk between them, rather than from intrinsic improvements in single‐junction conversion efficiency. Using low‐cost, HTL‐free mesoporous carbon PSCs with AVA‐MAPbI_3_, we demonstrate efficient and symmetrical bifacial performance under different indoor lighting conditions, simulating domestic (3000 K) and working (6500 K) environments.

Stability is ensured by the mutual encapsulation of the two PSCs. Control experiments performed under indoor ambient storage conditions demonstrate that the 2S‐SP PSC architecture provides improved durability compared with unencapsulated reference monofacial PSCs and same durability with respect to a passive‐glass encapsulated monofacial PSCs. We employed two different materials and deposition methods (laminated foil and drop‐casted polyurethane) to bond the two PSCs, obtaining comparable results in terms of performance durability. This allows for power retention of over 93% after 3500 h under ambient indoor conditions (25°C and 30–65% RH). These results confirm that the improved stability originates from the architectural configuration rather than from a specific encapsulation material. Edge sealing with PIB is not strictly needed but could be applied.

Indoor lighting simulations support the experimental observations by quantitatively demonstrating that the vertical two‐sided configuration increases the effective light intercepted by the device by simultaneously collecting direct, diffuse, and reflected light contributions typically present in indoor environments. These modelling results provide a physical framework linking device architecture to the measured performance enhancement.

The fabrication and use of a 2 × 2 2S‐SP mini‐module powering a sensing circuit confirms the viability of this architecture for real‐world, self‐powered IoT applications. This system‐level demonstration establishes the scalability of the concept and highlights its practical relevance for distributed indoor energy harvesting systems.

Scalability, performance, and robustness of the 2S‐SP PSC make them a competitive solution for next‐generation sustainable energy harvesting systems.

## 4 Experimental Section

### 4.1 mC‐PSCs Fabrication

The layers of monolithic architecture are screen‐printed on a fluorinedoped tin oxide glass substrate and consist of a compact titanium oxide (c‐TiO_2_) as hole blocking layer (~50 nm), a mesoporous TiO_2_ (mp‐TiO_2_) as electron transporting layer (~500 nm), a mesoporous zirconium oxide (ZrO_2_) as insulating layer (~1 μm), and a high temperature annealed carbon electrode (~10 mm). The entire stack is commercially available (Solaronix). For the fabrication of AVA‐MAPbI_3_ mC‐PSCs, we drop‐casted 5.76 µl of AVA‐MAPbI_3_ solution (Solaronix) on the mesoporous stack by using a micropipette. We waited 30 min to allow the full infiltration of the solution. Then, we anneal the samples at 60°C for 20 min to form the tetragonal photoactive phase. The active area of the device is 1.5 cm^2^.

### 4.2 mC‐PSCs Encapsulation

For the foil encapsulated 2S‐SP PSC, a commercial foil composed of a blend of polyolefin and paraffin wax is used between the two PSCs and vacuum laminated at 70°C for 2 min, with a layer of adhesive Kapton tape used as an interlayer. A cover glass is used in the reference device. For the polyurethane encapsulated devices, polyol and diisocyanate precursors were provided by Demak Polymers. Polyol resin with viscosity ∼1300 Cp is composed of 80% Sovermol 780, 20% Polyol 3610, and a 0.05% tin‐based catalyst, in weight. Diisocyanate resin with viscosity ∼1100 Cp is composed by 40% Tolonate X Flo 100 and 60% Desmodur Eco N7300 in weight. Aliphatic ether‐ester‐based polyurethane is obtained by mixing polyol and diisocyanate (1:2.5 weight ratio) for 5 min at RT. Prior to deposition, the mixture is subjected to a vacuum treatment to eliminate the air bubble and the humidity trapped during the mixing process. The PU mixture had a gel time of 45 min. PU is thus deposited by drop‐casting on device and covered by a bare glass (for reference mC‐PSC) or by another mC‐PSC (for the 2S‐SP PSC). Then the device is stored at RT and in a N_2_ environment for 3 days to allow the complete polymerization of the encapsulant layer avoiding intrusion of air humidity.

The contacts are brought on the rear side of the device through Ag paste (Solaronix Elcosil).

### 4.3 Device Characterization under 1 Sun

J–V characteristics were measured in ambient air by a digital source meter (Keithley model 2401) under AM 1.5‐simulated sunlight (100 mW/cm^2^) from Peccell PEC‐L01. Scan range was from −0.1 to 1.2 V for forward scan and from 1.2 to −0.1 V for reverse scan with a step of 0.01 V with a scan rate of 200 mV/s. Stabilized power is measured by applying a constant voltage (corresponding to the MPP) and measuring the photocurrent.

### 4.4 Device Characterization Under Indoor Illumination

J–V characteristics were measured in ambient air by a digital source meter (Keithley model 2401) under 5 LED lamps of 6500 K or 3000 K. Power of the lamps is regulated to achieve different illuminance conditions. Measurements were performed inside a box of dimensions length x width x height 30x40x62 cm^3^ with black‐painted walls to avoid diffused light or white‐painted walls to simulate a real room.

### 4.5 Lamps Irradiance and Spectrum Measurements

A spectrophotometer, provided by Cicci Research s.r.l., was used to acquire the light spectrum of lamps. The measurements were carried out in a spectral range from 330 nm to 1100 nm with a spectral resolution of 1 nm and an integration time of 10 ms. The irradiance was measured by using a commercial Luxmeter (UNIKS E300).

### 4.6 System Integration IoT node

The perovskite module is connected to the STDES‐ISV002V1 board, a reference design based on ST's SPV1050 energy harvesting IC. This board efficiently manages and maximizes the energy harvested from the perovskite module by performing voltage regulation, energy storage, and protection functions, thereby providing a stable 1.8 V power supply to the IoT node. Optimized for low‐light indoor environments, the system ensures maximum energy harvesting efficiency while minimizing losses. The IoT node, developed as part of the Samothrace project, integrates high‐precision STTS22 temperature and HTS221 humidity sensors, along with additional sensing capabilities, to enable comprehensive indoor environmental monitoring. The entire system is engineered for ultralow power consumption, allowing autonomous operation with the energy available from the perovskite module.

### 4.7 Room Lighting Simulations

Dialux evo was used to perform lighting simulations. A typical office room was modelled (3.6 × 5.4 × 3.0 m^3^) with a mean horizontal illuminance > 500 Lx at desk height (75 cm) in all the positions where desks are located and with a ratio between mean illuminance and minimum illuminance on the working area > 0.6. Both values comply with European standard EN 12464‐1 of the Lighting of Workplaces for office requiring Illuminance_mean_ > 500 Lx and Illuminance_min_/ Illuminance_mean_ > 0.60 on working plane (desks). 2S‐SP module is supposed to be 20x20 cm^2^.

We simulated integration of 2S‐SP module on a corner of the room at a height of 240 cm from floor and on white office desk (90% reflectance. 75 cm from the floor).

## Supporting Information

Additional supporting information can be found online in the Supporting Information section. **Supporting Fig. S1:** Comparison of Power vs Voltage curves of PSC 1 and PSC 2 forming 2S‐SP PSC (connected in parallel) with mismatching voltage and with matching voltage individually illuminated by a vertical illuminance of 1800 lux (6500 K). As seen in the left panel, when two sub‐cells with different working voltages are connected in parallel, the total output (blue curve) is constrained by the sub‐cell with the lower operating voltage (black curve). In a parallel circuit, both devices are forced to operate at the same terminal voltage, which causes: 1)the higher‐voltage sub‐cell (red curve) to operate below its optimal maximum‐power point, 2) the lower‐voltage sub‐cell to dominate the overall I–V characteristic (black curve), and 3) a substantial reduction of the total power compared to the ideal sum of the two individual devices (blue curve). In the case of two cells illuminated by a vertical illuminance of 920 lux (6500 K) with matching working voltages (right panel), the combined output (blue curve) closely follows the expected parallel superposition of the two identical sub‐cells (black curve), with doubling of generated power, demonstrating minimal power loss. **Supporting Fig. S2:** (a) Rendering of the simulated room illuminated by three square LED panels (4000K) and horizontal illuminance at 240 cm above the floor. (b) Horizontal illuminance map (lux) at 240 cm above the floor. (c) Rendering showing the position of the 2S‐SP PSC in horizontal configuration, where horizontal illuminance values were calculated. (d) Rendering showing the position of the 2S‐SP PSC in vertical configuration, where vertical illuminance values were calculated, at a distance of 30 cm from the corner and at 45° angle with walls. (e) Table summarizing the horizontal and vertical illuminance on the two faces of the 2S‐SP PSC, the corresponding total vertical illuminance, and the illuminance gain of the vertical bifacial configuration relative to the horizontal monofacial configuration. **Supporting Fig. S3:** (a) Rendering of the simulated room illuminated by three square LED panels (4000K). (b) Horizontal illuminance map (lux) at 75 cm above the floor on desk. (c) Positions of the vertical 2S‐SP PSC oriented along the short axis of the room, where vertical and horizontal illuminance values were calculated. (d) Rendering of position A, showing the 2S‐SP PSC mounted on a white desk. (e) Table summarizing the horizontal and vertical illuminance on the two faces of the 2S‐SP PSC, the corresponding total vertical illuminance, and the illuminance gain of the vertical configuration relative to the horizontal monofacial configuration. **Supporting Fig. S4:** (a) Rendering of the simulated room illuminated by three square LED panels (4000K) and horizontal illuminance at 75 cm above the floor on desk. (b) Horizontal illuminance map (lux) at 80 cm above the floor. (c) Positions of the vertical 2S‐SP PSC oriented along the long axis of the room, where vertical and horizontal illuminance values were calculated. (d) Rendering of position A, showing the 2S‐SP PSC mounted on a white desk. (e) Table summarizing the horizontal and vertical illuminance on the two faces of the 2S‐SP PSC, the corresponding total vertical illuminance, and the illuminance gain of the vertical configuration relative to the horizontal monofacial configuration. **Supporting Fig. S5:** J‐V curves of PSC 1 and PSC 2 forming 2S‐SP PSC, before and after the encapsulation process. It causes a modest efficiency drop of ~6.5%, due to slight reductions in V_oc_ and fill factor, while J_sc_ shows a small increase. **Supporting Fig. S6:** Spectrum of the white LED bulb (6500 K). **Supporting Fig. S7:** Comparison of PSC J‐V curves while illuminating it from the carbon side (darkening the glass side to avoid reflections) and illuminating it from the glass side. The J–V curve under carbon‐side illumination looks like a dark curve (J = 0 at V = 0). This observation reinforces that light entering from the exposed glass side cannot escape the carbon layer (which act as a full absorber) and therefore cannot reach the second device. Thus, from an optical perspective, each device in 2S‐PSC architecture operates as an independent single‐junction carbon PSC. **Supporting Fig. S8:** Vertical illuminance of the LED lamps at different power inside the whitepainted walls box vs distance from the LED lamps (or from the floor). **Supporting Fig. S9:** Stabilized power statistics at different illuminance conditions (LED bulb 6500 K) of 4 different samples for monofacial and 2S‐SP PSC. **Supporting Fig. S10:** Stability of the 2S‐SP PSC generated power (V = 0.48 V). **Supporting Fig. S11:** Comparison of the J‐V curves, Maximum Power extracted from J‐V curved, and stabilized power at 0.48 V (V_MPP_) at different illuminance conditions (LED bulb 6500 K) in horizontal configuration for side A and side B of the 2S‐SP PSC. **Supporting Fig. S12:** Spectrum of the white LED bulb (3000 K). **Supporting Fig. S13:** Comparison of the J‐V curves, Maximum Power extracted from J‐V curved, and stabilized power at 0.48 V (V_MPP_) at different illuminance conditions (LED bulb 3000 K) in horizontal configuration for side A and side B of the 2S‐SP PSC. **Supporting Fig. S14:** Comparison of the J‐V curves (upper panel) and the stability over the time (bottom panel) in indoor conditions (30–65% RH and 25°C) of 2S‐SP PSC encapsulated with polyurethane resin and reference monofacial PSC. The power output slightly increases after 250 h and stabilizes up to at least 3500 h under continuous indoor conditions (30‐65% RH and 25°C), while the unencapsulated device efficiency monotonically decreases reaching ~80% of the initial efficiency. The lines are guides for the eyes. **Supporting Fig. S15:** Photo of the 2S‐SP mini‐module inside the box simulating indoor conditions (6500 K LED bulb ‐ 920 lux). **Supporting Fig. S16:** Detailed scheme of the simplified proof‐of‐concept circuit designed to power a board equipped with a humidity and temperature sensor. **Supporting Fig. S17:** Battery‐voltage evolution of the 2S‐SP mini‐module–battery system under alternating 1 h light / 1 h dark indoor illumination (920 lux, 6500 K). The stepwise increase in voltage during each light period and the minimal decrese of the voltage in the dark confirm stable charging behavior under intermittent lighting conditions. **Supporting Table S1:** Photovoltaic parameters of the two sides of the 2S‐SP PSC at different illuminance conditions (LED Bulb 6500 K). **Supporting Table S2:** Photovoltaic parameters of the 2S‐SP PSC at different illuminance conditions (LED Bulb 3000 K).

## Author Contributions

**Salvatore Valastro:** conceptualization (lead), data curation (lead), formal analysis (lead), investigation (lead), methodology (lead), writing – original draft (lead). **Sebastian Ferranti:** investigation (equal). **Rosa Previti:** data curation (equal), formal analysis (equal). **Michele Dellutri:** data curation (equal), formal analysis (equal). **Simone Galliano:** methodology (equal). **Alessandra Alberti:** funding acquisition (lead), investigation (lead), resources (lead), supervision (lead), validation (lead), writing – review and editing (lead).

## Funding

This work was supported by Ministero dell'Università e della Ricerca (Grant CUP B63C22000620005), Ministero dell'ambiente e della sicurezza energetica (Grant CUP B53C22005670005)

## Conflict of Interest

The authors declare no conflicts of interest.

## Supporting information

Supplementary Material

## Data Availability

The data that support the findings of this study are available from the corresponding author upon reasonable request.
