# Supplementary material for: Design and Performance of Two‐Sided Self‐Protecting Perovskite Solar Cells for Indoor Vertical Applications
Source: Small Sci. 2026 Mar 19;6(3):e202500603. doi: 10.1002/smsc.202500603 (PMC13097510; doi:10.1002/smsc.202500603)
Supplement: Supplementary file 1 — Supplementary Material [file SMSC-6-e202500603-s001.pdf]

Supporting information

Design and Performance of Two-sided Self-Protecting Perovskite Solar Cells for Indoor Vertical Applications

Salvatore Valastro[1], Sebastian Ferranti[1], Rosa Previti[2], Michele Dellutri[2], Simone Galliano[3], Alessandra Alberti[1]*

[1]*CNR-IMM, Zona Industriale strada VIII n°5, 95121, Catania, Italy*
[2] *STMicroelectronics Srl, Stradale Primosole 50, 95121, Catania, Italy*
[3] *Department of Chemistry, NIS Interdepartmental Centre and INSTM Reference Centre, University of Turin, Turin 10125, Italy*

*salvatore.valastro@cnr.it

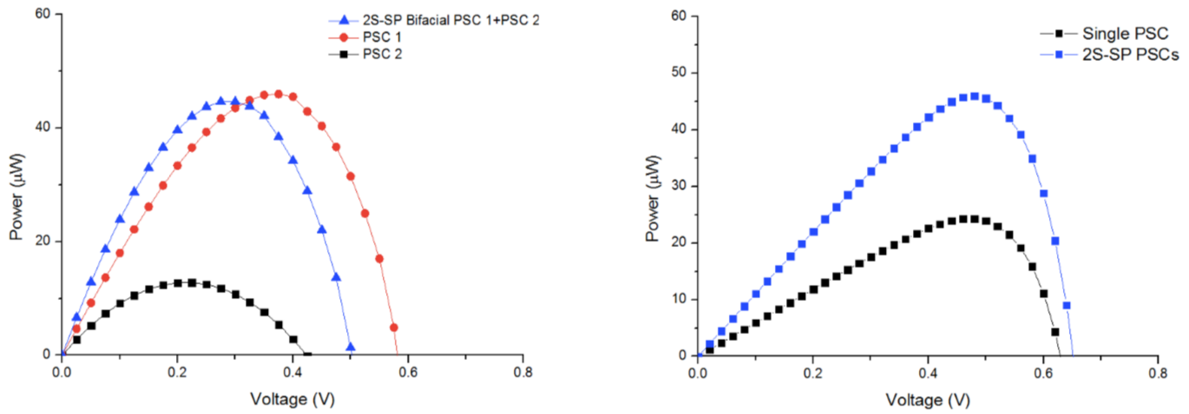

Figure S1 Comparison of Power vs Voltage curves of PSC 1 and PSC 2 forming 2S-SP PSC (connected in parallel) with mismatching voltage and with matching voltage individually illuminated by a vertical illuminance of 1800 lux (6500 K). As seen in the left panel, when two sub-cells with different working voltages are connected in parallel, the total output (blue curve) is constrained by the sub-cell with the lower operating voltage (black curve). In a parallel circuit, both devices are forced to operate at the same terminal voltage, which causes: 1)the higher-voltage sub-cell (red curve) to operate below its optimal maximum-power point, 2) the lower-voltage sub-cell to dominate the overall I–V characteristic (black curve), and 3) a substantial reduction of the total power compared to the ideal sum of the two individual devices (blue curve). In the case of two cells illuminated by a vertical illuminance of 920 lux (6500 K) with matching working voltages (right panel), the combined output (blue curve) closely follows the expected parallel superposition of the two identical sub-cells (black curve), with doubling of generated power, demonstrating minimal power loss.

(a) (b) (c) (d)

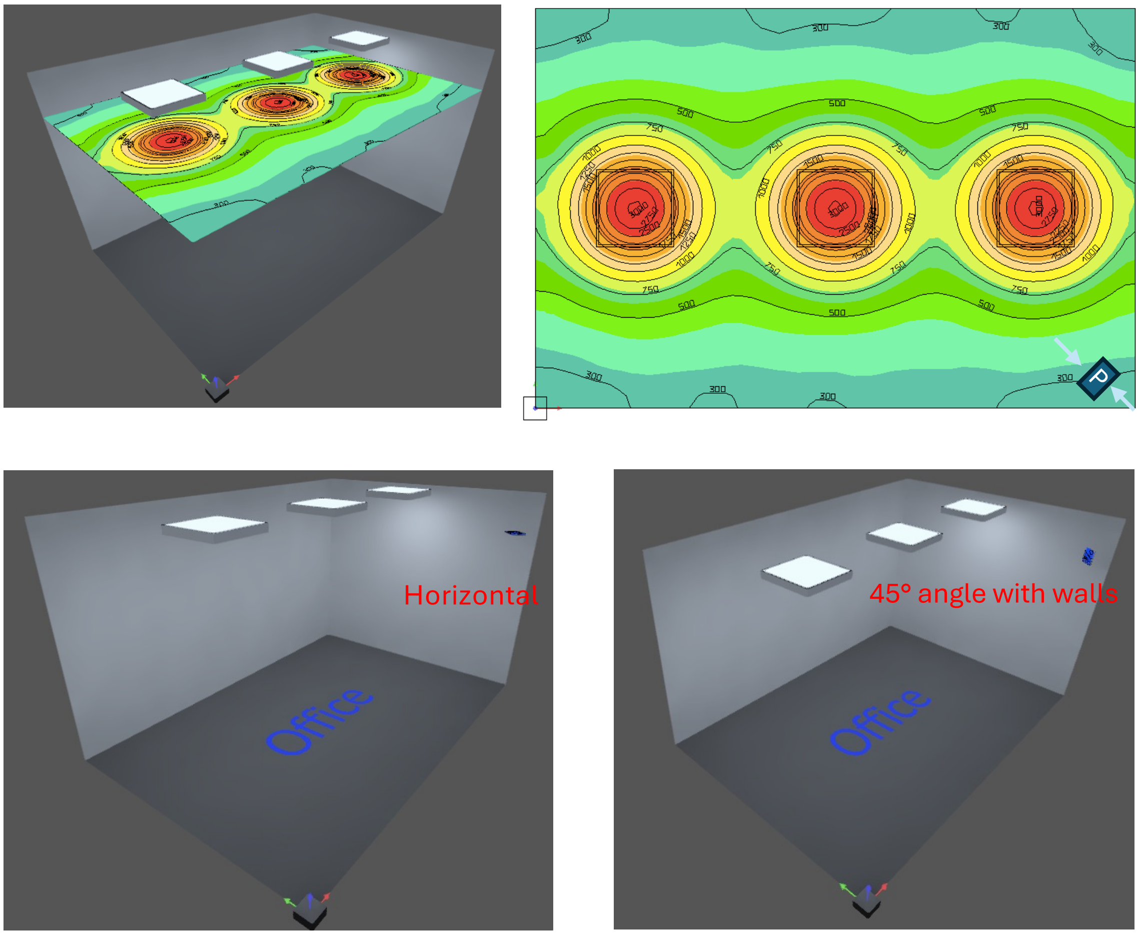

(e)

	Horizontal Illuminance Light side (Lux)	Horizontal Illuminance Floor side (Lux)	Vertical Illuminance Light Side (Lux)	Vertical Illuminance Wall Side (Lux)	Vertical Illuminance Total (Lux)	Gain (%)
Position P 45° With Walls	274	272	364	264	628	129

Figure S2 (a) Rendering of the simulated room illuminated by three square LED panels (4000K) and horizontal illuminance at 240 cm above the floor. (b) Horizontal illuminance map (lux) at 240 cm above the floor. (c) Rendering showing the position of the 2S-SP PSC in horizontal configuration, where horizontal illuminance values were calculated. (d) Rendering showing the position of the 2S-SP PSC in vertical configuration, where vertical illuminance values were calculated, at a distance of 30 cm from the corner and at 45° angle with walls. (e) Table summarizing the horizontal and vertical illuminance on the two faces of the 2S-SP PSC, the corresponding total vertical illuminance, and the illuminance gain of the vertical bifacial configuration relative to the horizontal monofacial configuration.

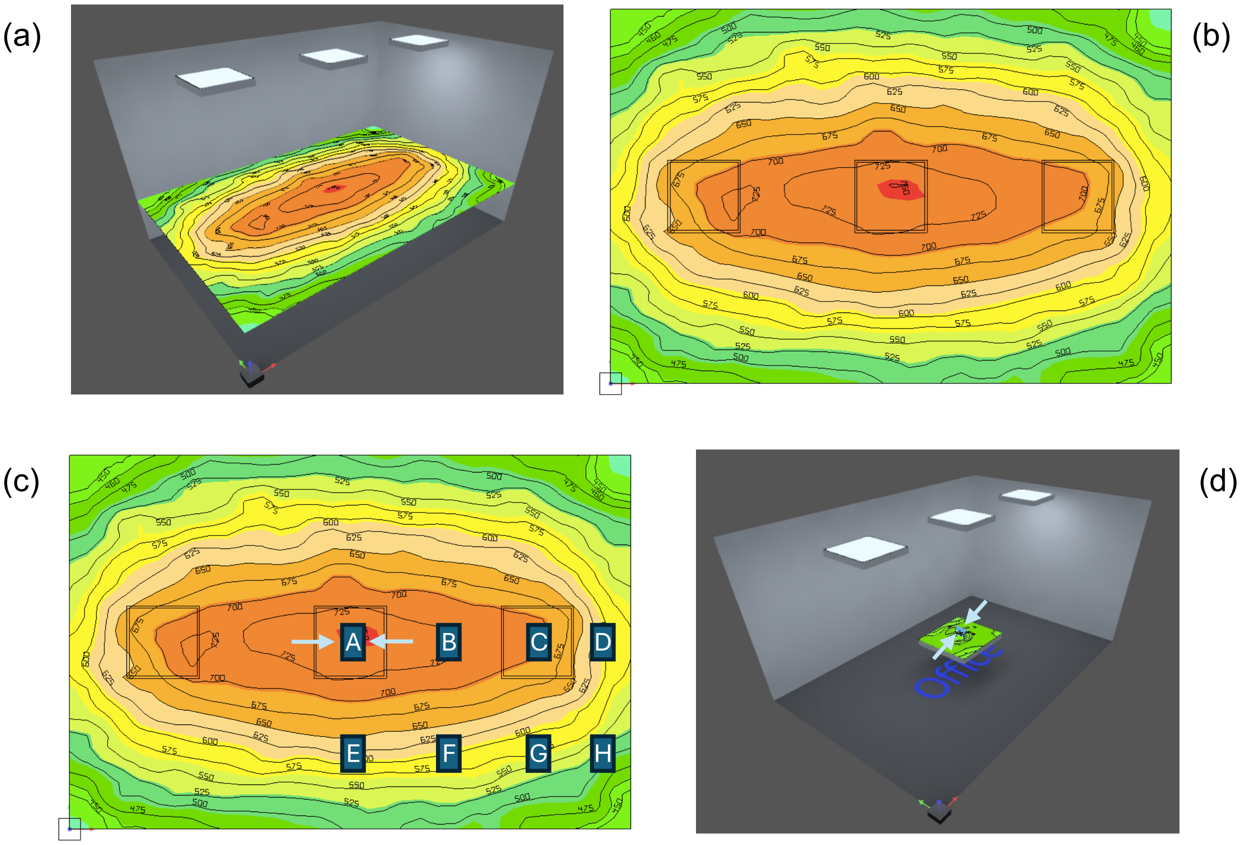

(e)

	Horizontal Illuminance on desk (Lux)	Vertical Illuminance Left Side (Lux)	Vertical Illuminance Right Side (Lux)	Vertical Illuminance Total (Lux)	Gain (%)
Position A	750	493	491	984	31.2
Position B	725	523	494	1017	40.3
Position C	700	498	431	929	32.7
Position D	600	514	407	921	53.5
Position E	600	439	440	879	46.5
Position F	575	464	436	900	56.5
Position G	550	443	381	824	49.8
Position H	525	446	372	818	55.8

Figure S3 (a) Rendering of the simulated room illuminated by three square LED panels (4000K). (b) Horizontal illuminance map (lux) at 75 cm above the floor on desk. (c) Positions of the vertical 2S-SP PSC oriented along the short axis of the room, where vertical and horizontal illuminance values were calculated. (d) Rendering of position A, showing the 2S-SP PSC mounted on a white desk. (e) Table summarizing the horizontal and vertical illuminance on the two faces of the 2S-SP PSC, the corresponding total vertical illuminance, and the illuminance gain of the vertical configuration relative to the horizontal monofacial configuration.

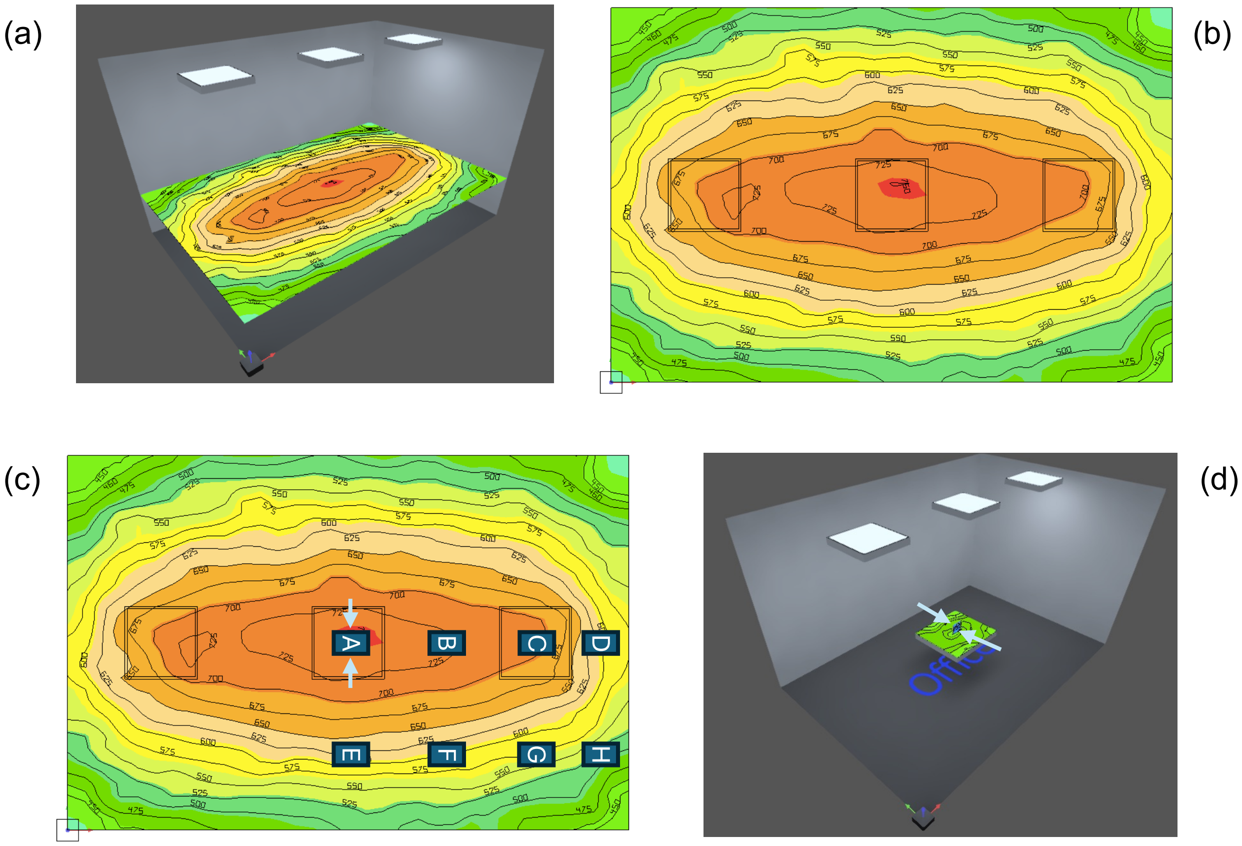

(e)

	Horizontal Illuminance on desk (Lux)	Vertical Illuminance Up Side (Lux)	Vertical Illuminance Bottom Side (Lux)	Vertical Illuminance Total (Lux)	Gain (%)
Position A	750	426	427	853	13.7
Position B	725	423	422	845	16.6
Position C	700	414	414	828	18.3
Position D	600	402	403	805	34.2
Position E	600	524	386	910	51.7
Position F	575	519	383	902	56.9
Position G	550	493	366	859	56.2
Position H	525	476	360	836	59.2

Figure S4 (a) Rendering of the simulated room illuminated by three square LED panels (4000K) and horizontal illuminance at 75 cm above the floor on desk. (b) Horizontal illuminance map (lux) at 80 cm above the floor. (c) Positions of the vertical 2S-SP PSC oriented along the long axis of the room, where vertical and horizontal illuminance values were calculated. (d) Rendering of position A, showing the 2S-SP PSC mounted on a white desk. (e) Table summarizing the horizontal and vertical illuminance on the two faces of the 2S-SP PSC, the corresponding total vertical illuminance, and the illuminance gain of the vertical configuration relative to the horizontal monofacial configuration.

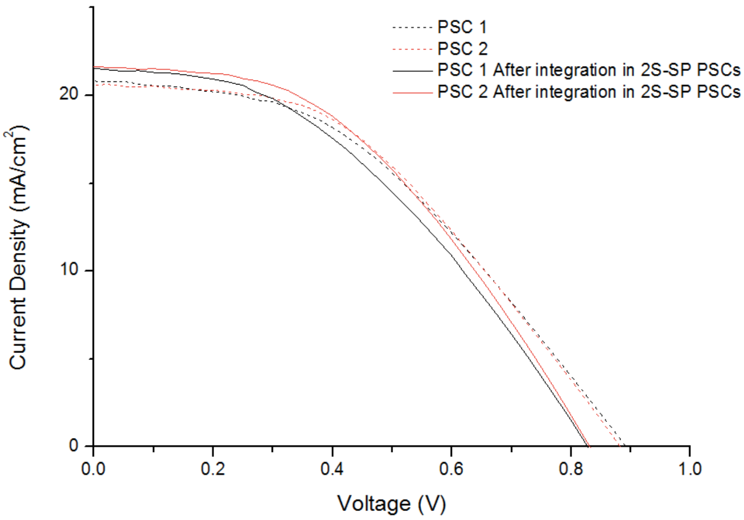

Figure S5 J-V curves of PSC 1 and PSC 2 forming 2S-SP PSC, before and after the encapsulation process. It causes a modest efficiency drop of ~6.5%, due to slight reductions in Voc and fill factor, while Jsc shows a small increase.

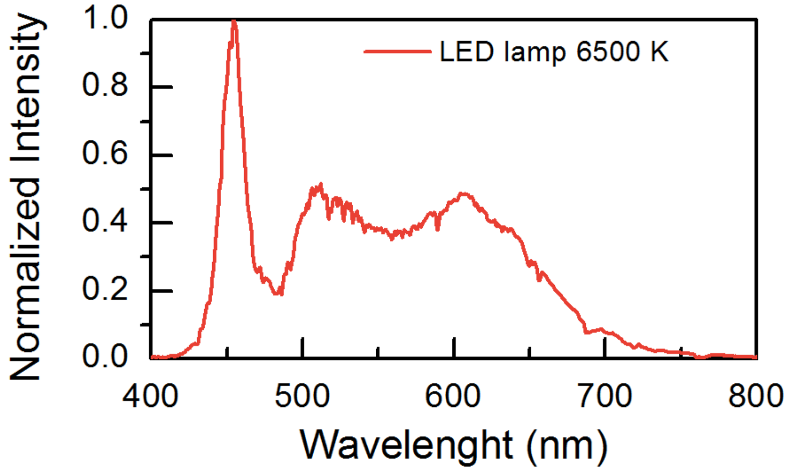

Figure S6 Spectrum of the white LED bulb (6500 K)

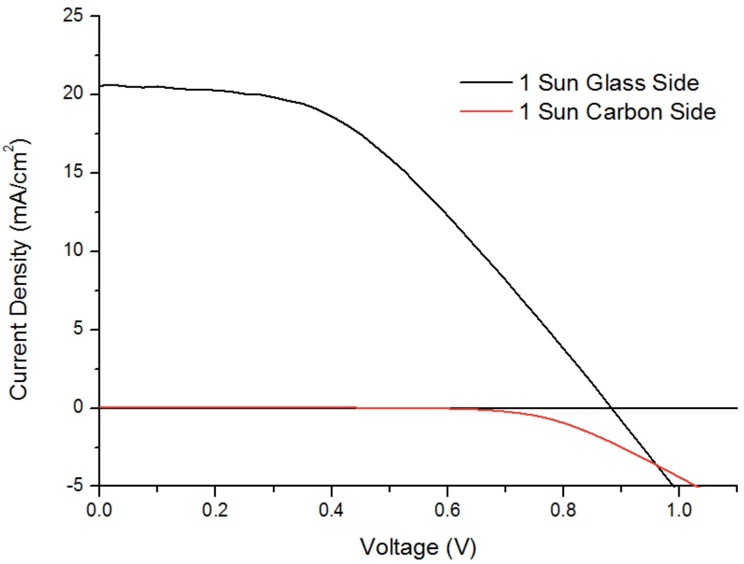

Fig. S7 Comparison of PSC J-V curves while illuminating it from the carbon side (darkening the glass side to avoid reflections) and illuminating it from the glass side. The J–V curve under carbon-side illumination looks like a dark curve (J=0 at V=0). This observation reinforces that light entering from the exposed glass side cannot escape the carbon layer (which act as a full absorber) and therefore cannot reach the second device. Thus, from an optical perspective, each device in 2S-PSC architecture operates as an independent single-junction carbon PSC.

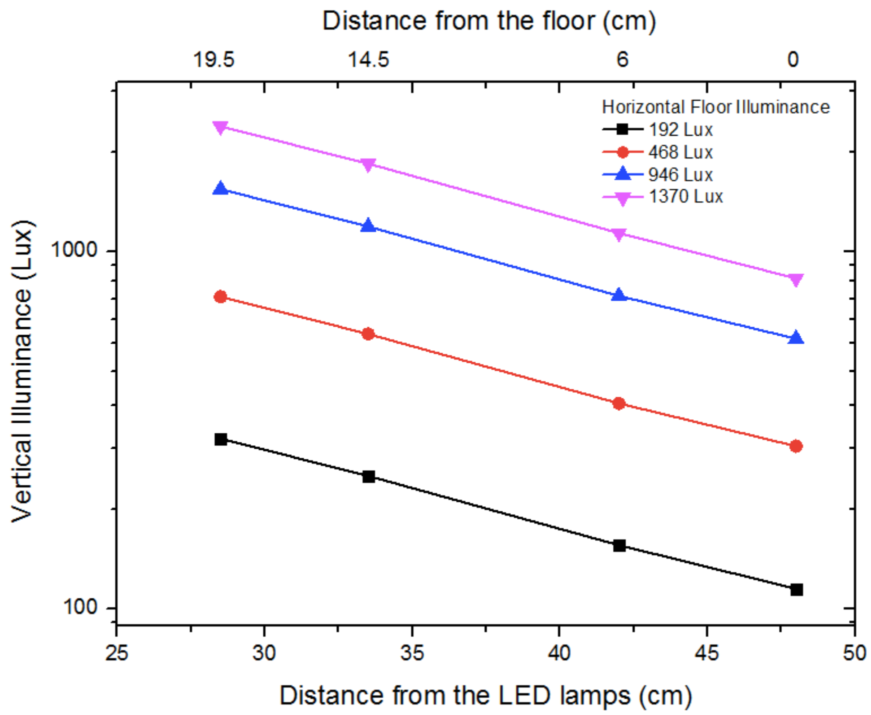

Figure S8 Vertical illuminance of the LED lamps at different power inside the white-painted walls box vs distance from the LED lamps (or from the floor)

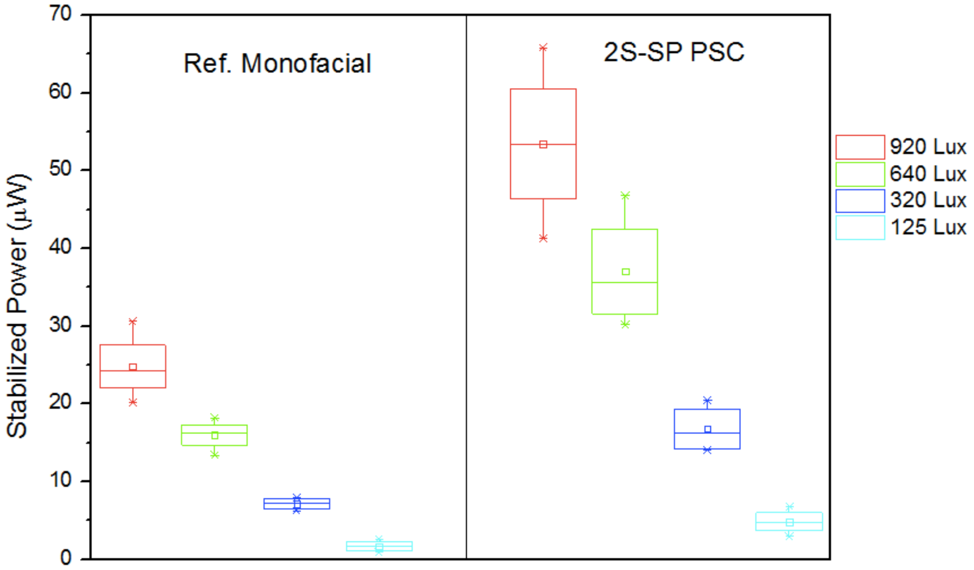

Figure S9 Stabilized power statistics at different illuminance conditions (LED bulb 6500 K) of 4 different samples for monofacial and 2S-SP PSC.

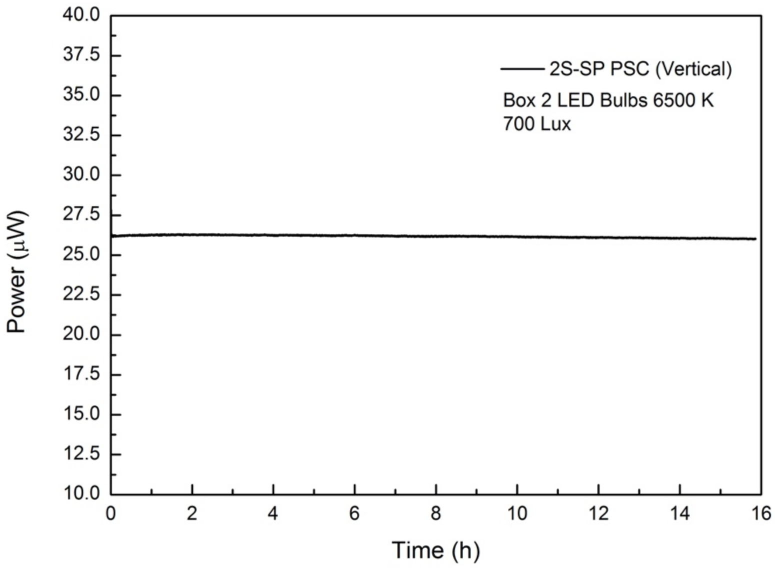

Figure S10 Stability of the 2S-SP PSC generated power (V=0.48 V)

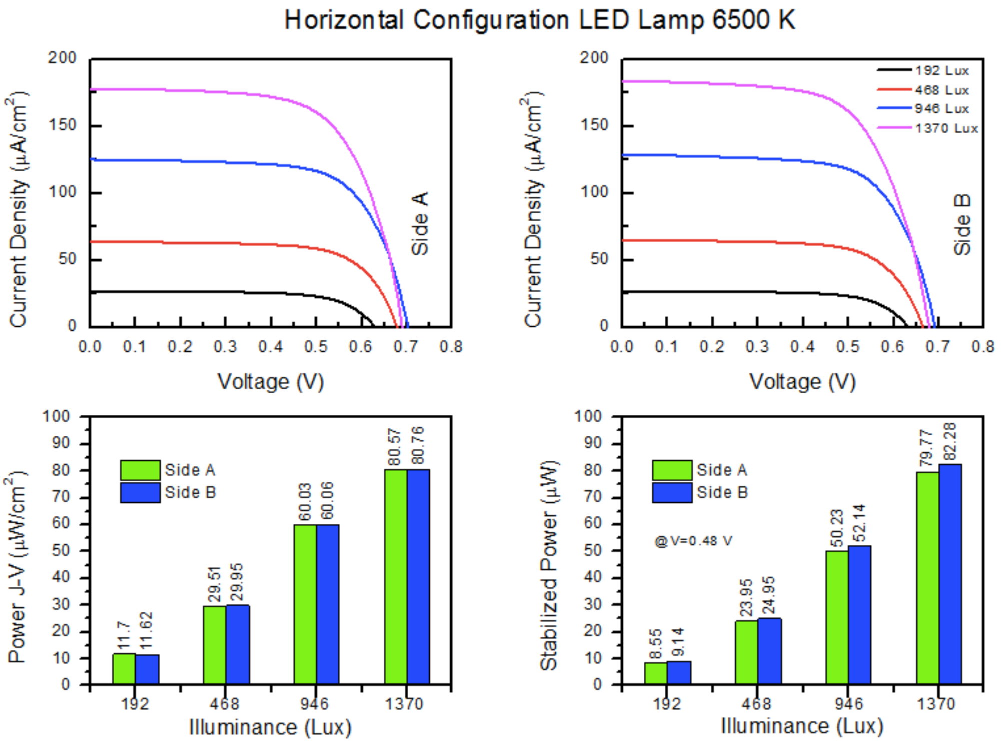

Figure S11 Comparison of the J-V curves, Maximum Power extracted from J-V curved, and stabilized power at 0.48 V (V_{MPP}) at different illuminance conditions (LED bulb 6500 K) in horizontal configuration for side A and side B of the 2S-SP PSC

Illuminance 6500 K Led Bulb (Lux)	192	468	946	1372
Measured Irradiance (μW/cm2)	0.65	1.58	3.21	4.65
Stabilized Power Side A @0.48 V (μW)	8.91	27.46	57.92	83.93
Stabilized Power Side B @0.48 V (μW)	9.31	28.51	59.78	87.07
Voc Side A (V)	0.63	0.68	0.70	0.69
Voc Side B (V)	0.63	0.67	0.70	0.68
Jsc Side A (μA/cm2)	26.8	63.9	125.3	178.1
Jsc Side B (μA/cm2)	27.0	65.3	128.8	183.6
Fill factor Side A (%)	69.3	67.9	68.4	65.6
Fill factor Side B (%)	68.3	68.5	66.6	64.7
Maximum Power from JV Side A (μW/cm2)	11.70	29.51	60.03	80.57
Maximum Power from JV Side B (μW/cm2)	11.62	29.95	60.06	80.76
Stabilized PCE Side A @0.48 V (%)	7.99	11.62	11.97	12.08
Stabilized PCE Side B @0.48 V (%)	8.34	12.06	12.36	12.53
PCE from JV Side A (%)	17.98	18.62	18.73	17.33
PCE from JV Side B (%)	17.86	18.90	18.74	17.38

Table S1 Photovoltaic parameters of the two sides of the 2S-SP PSC at different illuminance conditions (LED Bulb 6500 K)

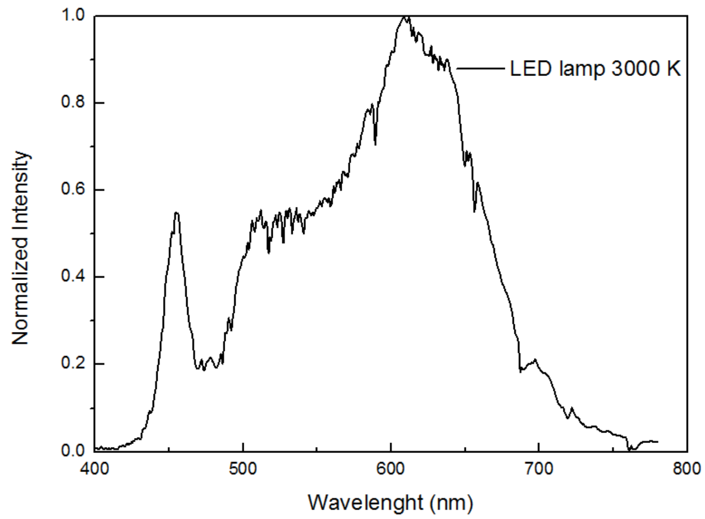

Figure S12 Spectrum of the white LED bulb (3000 K)

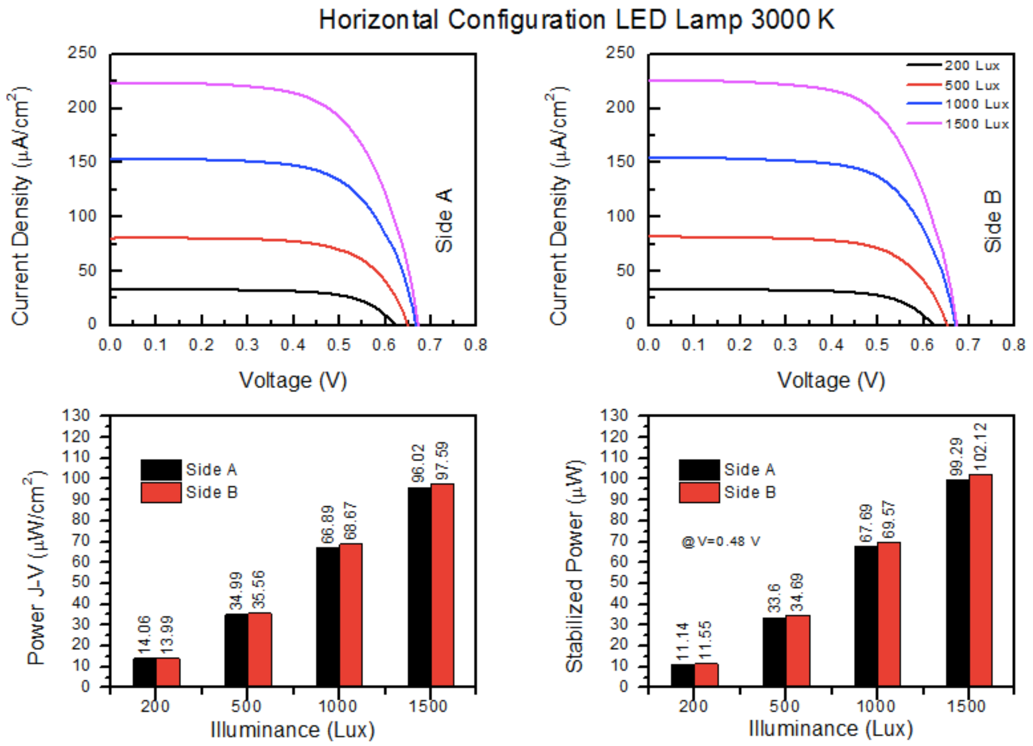

Figure S13 Comparison of the J-V curves, Maximum Power extracted from J-V curved, and stabilized power at 0.48 V (V_{MPP}) at different illuminance conditions (LED bulb 3000 K) in horizontal configuration for side A and side B of the 2S-SP PSC

Illuminance 3000 K Led Bulb (Lux)	200	500	1000	1500
Measured Irradiance (μW/cm2)	0.65	1.82	3.59	5.27
Stabilized Power Side A @0.48 V (μW)	11.1	33.6	67.7	99.3
Stabilized Power Side B @0.48 V (μW)	11.6	34.7	69.6	102.1
Voc Side A (V)	0.62	0.65	0.67	0.67
Voc Side B (V)	0.62	0.65	0.67	0.67
Jsc Side A (μA/cm2)	32.8	80.6	153.5	223.7
Jsc Side B (μA/cm2)	33.1	81.9	155.1	226.2
Fill factor Side A (%)	69.3	66.8	65.0	64.1
Fill factor Side B (%)	68.2	66.9	66.1	64.4
Maximum Power from JV Side A (μW/cm2)	14.1	35.0	66.9	96.0
Maximum Power from JV Side B (μW/cm2)	14.0	35.6	68.7	97.6
Stabilized PCE Side A @0.48 V (%)	11.4	12.3	12.6	12.6
Stabilized PCE Side B @0.48 V (%)	11.8	12.7	12.9	12.9
PCE from JV Side A (%)	21.6	19.2	18.6	18.2
PCE from JV Side B (%)	21.4	19.5	19.1	18.5

Table S2 Photovoltaic parameters of the 2S-SP PSC at different illuminance conditions (LED Bulb 3000 K)

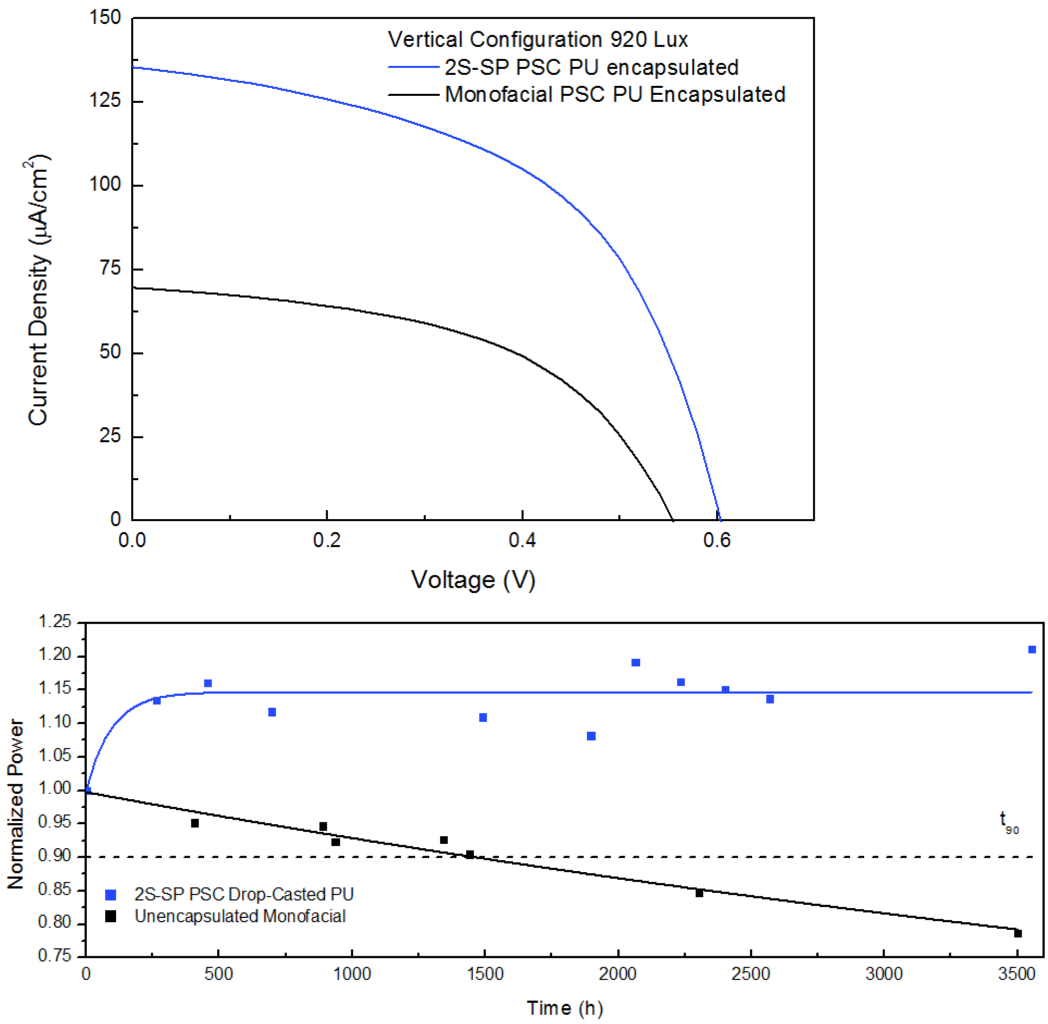

Figure S14 Comparison of the J-V curves (upper panel) and the stability over the time (bottom panel) in indoor conditions (30-65% RH and 25 °C) *of 2S-SP PSC encapsulated with polyurethane resin and reference monofacial PSC. The power output slightly increases after 250 h and stabilizes up to at least 3500 h under continuous indoor conditions (30-65% RH and 25 °C), while the unencapsulated device efficiency monotonically decreases reaching ~80% of the initial efficiency. The lines are guides for the eyes.*

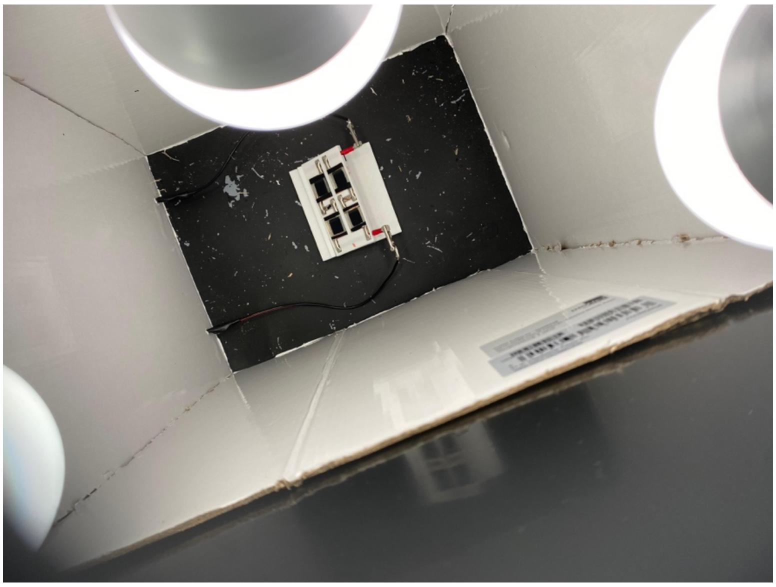

Figure S15 Photo of the 2S-SP mini-module inside the box simulating indoor conditions (6500 K LED bulb - 920 lux)

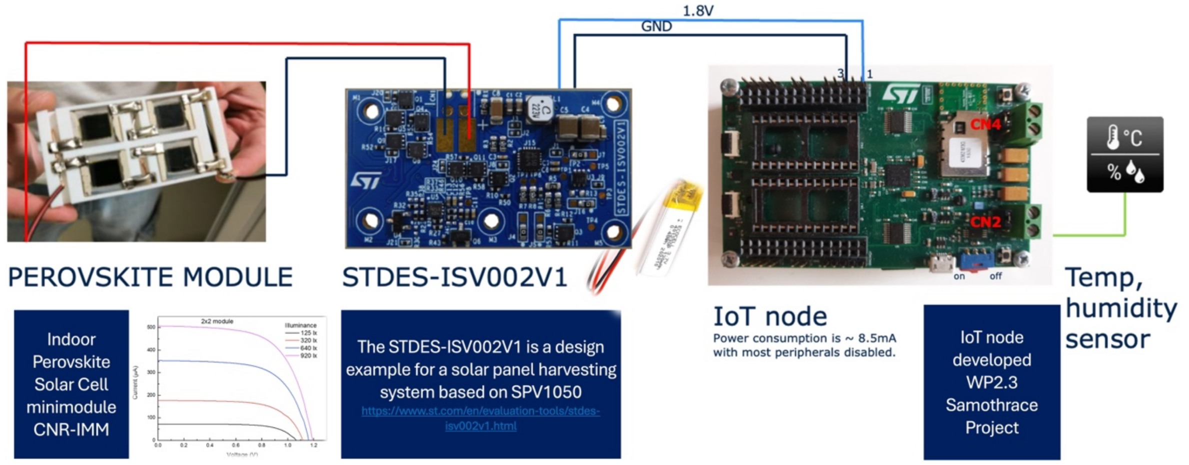

Figure S16 Detailed scheme of the simplified proof-of-concept circuit designed to power a board equipped with a humidity and temperature sensor.

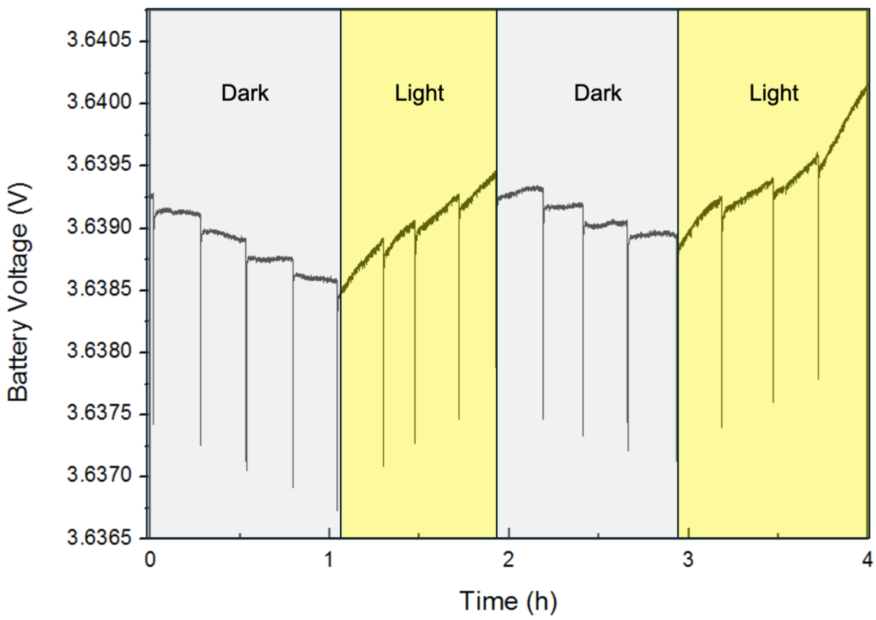

Figure S17 Battery-voltage evolution of the 2S-SP mini-module–battery system under alternating 1 h light / 1 h dark indoor illumination (920 lux, 6500 K). The stepwise increase in voltage during each light period and the minimal decrese of the voltage in the dark confirm stable charging behavior under intermittent lighting conditions.
